# Extracellular Vesicles From Auditory Cells as Nanocarriers for Anti-inflammatory Drugs and Pro-resolving Mediators

**DOI:** 10.3389/fncel.2019.00530

**Published:** 2019-11-29

**Authors:** Gilda M. Kalinec, Lucy Gao, Whitaker Cohn, Julian P. Whitelegge, Kym F. Faull, Federico Kalinec

**Affiliations:** ^1^Department of Head and Neck Surgery, David Geffen School of Medicine, University of California, Los Angeles, Los Angeles, CA, United States; ^2^Pasarow Mass Spectrometry Laboratory, Department of Psychiatry and Biobehavioral Sciences, Jane and Terry Semel Institute for Neuroscience and Human Behavior, David Geffen School of Medicine, University of California, Los Angeles, Los Angeles, CA, United States

**Keywords:** extracellular vesicles, HEI-OC1 cells, cochlear inflammation, pro-resolving mediators, drug nanocarriers, intracochlear drug delivery

## Abstract

Drug- and noise-related hearing loss are both associated with inflammatory responses in the inner ear. We propose that intracochlear delivery of a combination of pro-resolving mediators, specialized proteins and lipids that accelerate the return to homeostasis by modifying the immune response rather than by inhibiting inflammation, might have a profound effect on the prevention of sensorineural hearing loss. However, intracochlear delivery of such agents requires a reliable and effective method to convey them, fully active, directly to the target cells. The present study provides evidence that extracellular vesicles (EVs) from auditory HEI-OC1 cells may incorporate significant quantities of anti-inflammatory drugs, pro-resolving mediators and their polyunsaturated fatty acid precursors as cargo, and potentially could work as carriers for their intracochlear delivery. EVs generated by HEI-OC1 cells were divided by size into two fractions, small (≤150 nm diameter) and large (>150 nm diameter), and loaded with aspirin, lipoxin A4, resolvin D1, and the polyunsaturated fatty acids (PUFA) arachidonic, eicosapentaenoic, docosahexanoic, and linoleic. Bottom-up proteomics revealed a differential distribution of selected proteins between small and large vesicles. Only 17.4% of these proteins were present in both fractions, whereas 61.5% were unique to smaller vesicles and only 3.7% were exclusively found in the larger ones. Importantly, the pro-resolving protein mediators Annexin A1 and Galectins 1 and 3 were only detected in small vesicles. Lipidomic studies, on the other hand, showed that small vesicles contained higher levels of eicosanoids than large ones and, although all of them incorporated the drugs and molecules investigated, small vesicles were more efficiently loaded with PUFA and the large ones with aspirin, LXA4 and resolvin D1. Importantly, our data indicate that the vesicles contain all necessary enzymatic components for the *de novo* generation of eicosanoids from fatty acid precursors, including pro-inflammatory agents, suggesting that their cargo should be carefully tailored to avoid interference with their therapeutic purpose. Altogether, these results support the idea that both small and large EVs from auditory HEI-OC1 cells could be used as nanocarriers for anti-inflammatory drugs and pro-resolving mediators.

## Introduction

Drug- and noise-related hearing loss (DRHL and NRHL, respectively) are intimately associated with inflammatory responses in the inner ear (Kaur et al., 2016; Lowthian et al., 2016; Kalinec et al., 2017; Keithley, 2018). Inflammation, a normal biological reaction aimed at restoring tissue and organ functionality and homeostasis, is usually divided into two phases: initiation and resolution (Kumar et al., 2014). The initiation of inflammation is characterized by the up-regulation of pro-inflammatory mediators such as leukotrienes, prostaglandins, and thromboxanes. When the inflammatory response peaks, the resolution phase starts. Inflammatory resolution is an active process achieved mostly by the action of specialized protein and lipid pro-resolving mediators (Perretti, 2015; Perretti et al., 2015; Kalinec et al., 2017). We have recently proposed that stimulation of pro-resolving pathways associated with cochlear inflammatory processes could be an important new therapeutic approach for preventing or ameliorating DRHL and NRHL (Kalinec et al., 2017), with pro-resolving mediators accelerating the return to homeostasis by modifying the immune response rather than by inhibiting inflammation (Dalli and Serhan, 2019). The successful implementation of this clinical strategy, however, has several challenges. It requires, for instance, identifying safe and efficient ways to deliver a combination of pro-resolving mediators into the cochlea in clinically significant amounts, without compromising their pharmacokinetics and therapeutic efficacy and with minimal adverse effects to the host. Thus, this study was aimed at evaluating whether extracellular vesicles (EVs) from auditory HEI-OC1 cells could be adequate carriers for these agents.

Recent studies have shown that packaging drugs into nanoscale synthetic particles can improve pharmacokinetic efficiency and therapeutic efficacy (Li et al., 2017; Hao and Li, 2019). Nonetheless, the use of these drug-loaded artificial nanoparticles has several disadvantages. For example, microparticles are usually toxic, they are not stable in biological environments, assembling them is usually expensive, and they are not well suited to carry several different drugs simultaneously (Tang et al., 2012). EVs, in contrast, are naturally adapted for conveying molecular products from cells that generate and/or store them to cells that need them. After dexamethasone treatment, for example, Hensen cells in the guinea pig organ of Corti accumulate Annexin A1 in their cytoplasm and release it to the external milieu inside EVs (Kalinec et al., 2009). EVs have demonstrated promise as a natural delivery system for combinations of small molecules, proteins, oligonucleotides, and pharmacological drugs (Robbins and Morelli, 2014; Armstrong and Stevens, 2018).

EVs have unique advantages as carriers to deliver drugs, such as their ability to overcome natural biological barriers and their intrinsic cell targeting properties. Besides, since EVs are formed from cellular membranes, they are not toxic and they can be easily manipulated for drug-packaging without restrictions associated with the physicochemical properties of drugs. Furthermore, since EVs do not self-replicate, they lack endogenous tumor-formation potential. Importantly, EVs are known to be effective for gene and drug delivery, and their surface and cargo can be engineered to target specific cell types and to deliver specific components (Marcus and Leonard, 2013). Moreover, encapsulating some pharmacological drugs into EVs reduce their toxicity (Tang et al., 2012). Finally, EVs can be produced in a scaled manner more easily than other therapeutics (Lai et al., 2019), and because of their small size and scarce presence of membrane histocompatibility molecules, they carry a reduced possibility for immune rejection (Reis et al., 2016). Not less important, intracochlear delivery of EVs loaded with adeno-associated virus has been already successfully used for rescuing hearing in a mouse model for hereditary deafness (György et al., 2017).

EVs are released by cells as self-contained vesicles encapsulating a small portion of the parent cell cytoplasm. Cells naturally produce a diverse spectrum of EVs, spanning from small vesicles of about 50 nm diameter, to large vesicles up to 10 μm diameter (Mathieu et al., 2019). In the 1980s the smallest EV, between 50 and 150 nm in diameter, were called “exosomes,” and this term rapidly became the most frequently used in the EV field (Tkach et al., 2018); the bigger EVs are known as “ectosomes” or microvesicles. This categorization is also associated with the specific origin of the vesicles, with exosomes deriving from the fusion of multivesicular endosomes with the plasma membrane and microvesicles shedding directly from the plasma membrane (Colombo et al., 2014). Independently of their origin, EVs consist of a lipid bilayer, with integral and surface proteins, and containing in the core cytoplasmic proteins, lipids, RNAs (including messenger RNA and microRNAs), DNA, and metabolites (Pinheiro et al., 2018). They are usually enriched in proteins involved in vesicle genesis and trafficking, signal transduction, cytoskeleton organization, antigen presentation and transport, and vesicle targeting to acceptor cells or to the extracellular matrix (Robbins and Morelli, 2014). Typically, they also contain proteins belonging to the tetraspanin protein family such as CD9, CD63 and CD81. The lipid components of EVs include ceramide (sometimes used to differentiate them from lysosomes), cholesterol, sphingolipids, and phosphoglycerides with long and saturated fatty-acyl chains (Skotland et al., 2017). Importantly, it has been reported that EVs naturally contain lipid mediators such as arachidonic and 12-hydroxyeicosatetraenoic (12-HETE) acids, leukotriene B4, leukotriene C4, and prostaglandins E2 and J2 (PGE2 and 15d-PGJ2, respectively), as well as lipid-related proteins (Record, 2018). The outer surface of EVs, in turn, is rich in mannose, polylactosamine, α-2,6 sialic acid, N-linked glycans, lectins, and galectins (Laulagnier et al., 2004; Yanez-Mo et al., 2015). Nonetheless, each type of EV has a unique molecular signature that depends on the parent cell lineage and status (for example, healthy or pathological), and the stimulus that elicited their generation and release.

There is no actual proof yet that small EVs have a function different to that of larger EVs (Tkach et al., 2018; Vagner et al., 2019), although there is agreement in that these different EV populations may have different composition even when released by the same cell type (Mathivanan et al., 2010; Bobrie et al., 2012; Romancino et al., 2013; Zaborowski et al., 2015; Kowal et al., 2016; Xu et al., 2016; Vagner et al., 2019). A recent “consensus” publication, co-authored by several prominent scientists in the field of EVs, stated that “EVs in the small size range likely represent vesicles heterogeneous in origin” with unknown portions of exosomes and ectosomes (Mateescu et al., 2017). The definition of larger EVs is even less precise, with these vesicles comprising a wide range of membrane-enclosed entities. Indeed, it is not yet clear how to divide EVs into their relevant subtypes, or even how many functionally distinct subtypes there are (Mateescu et al., 2017). In addition, increasing evidence suggests that different classes of EVs, and different populations within each class, may harbor unique molecular cargos and have specific functions (Kowal et al., 2016; Willms et al., 2016; Jeppesen et al., 2019), clearly indicating the limitations of the size-based classification. Thus, looking for a better characterization, EVs have been divided by size into small and large (Tkach et al., 2018; Vagner et al., 2019). A similar approach was followed in the present study, with HEI-OC1 EVs divided into two fractions: small EVs (S-EVs, ≤150 nm in diameter, mostly exosomes), and large EVs (L-EVs, >150 nm in diameter, mostly microvesicles).

In summary, endogenously produced EVs are an attractive drug delivery system mainly because of their small size, low immunogenicity, absence of toxic effects, and stability in biological environments (Akao et al., 2011; Fuhrmann et al., 2015; Wong et al., 2016). In addition, EVs can be loaded with a designed combination of therapeutic agents and engineered to target specific cell types (Marcus and Leonard, 2013). Although the actual suitability of EVs for intracochlear delivery of anti-inflammatory and pro-resolving agents, as well as its true potential for preventing or alleviating DRHL and NRHL, will require additional research, confirming them as a possible safe and efficient nanocarrier for these agents is a crucial initial step. In the present study, we investigate the production of EVs by auditory HEI-OC1 cells, characterize them by proteomics and targeted lipidomics, and evaluate their capability to incorporate simultaneously anti-inflammatory and pro-resolving agents in clinically significant quantities. Our results support the idea that EVs from HEI-OC1 cells could, indeed, be used as nanocarriers for intracochlear delivery of drugs and molecular mediators aimed at facilitating the resolution of inflammatory processes.

## Materials and Methods

### HEI-OC1 Cells

Immortomouse^TM^-derived HEI-OC1 cells were grown in polystyrene cell culture dishes (CellStar^TM^, Greiner Bio-One, NC, USA) using DMEM (Gibco, Gaithersburg, MD, USA) supplemented with 10% FBS (HyClone, Thermo Fisher Scientific, Waltham, MA, USA), at 33°C and 10% CO_2_ as previously described (Kalinec et al., 2003). At approximately 80% confluence, cells were washed with PBS and fresh cell culture media supplemented with 10% exosome-depleted FBS (Cat. #A2720801, Thermo Fisher Scientific, Waltham, MA, USA) was added. After a further 24 h under the same incubation conditions, the cell culture media was removed and processed for EV isolation and characterization.

### EVs Isolation and Physical Characterization

EVs were isolated from the cell culture media using the commercially available exoEasy^TM^ isolation kit (Qiagen) following the manufacturer-suggested procedure. Two cell culture dishes of 100 mm diameter were used per condition, yielding 16 ml (8 ml/dish) of medium; this medium was pre-filtered to exclude particles larger than 800 nm, and the filtrate loaded onto the exoEasy^TM^ spin columns (4 ml per column, total four columns per condition). After centrifugation (500× *g*, 5 min) to remove the residual liquid, 1 ml of elution buffer was added to each column, and then centrifuged again for 5 min at 500× *g* to collect the eluate. Isolated EV samples (1 ml each in elution buffer per column) were pooled, PBS was added to obtain a final volume of 200 ml, and then this EV suspension was filtered by tangential flow with a Sartorious Vivaflow 50 device with a 200 nm pore size (Cat. #VF05P7. Sartorious GmbH, Göttingen, Germany). This device allows the recovery of two fractions: the retained one (L-EVs) with particles of diameter greater than 200 nm, and the filtrate (S-EVs) with particles smaller than 200 nm diameter. The L-EVs fraction was further ultra-filtered and concentrated using Sartorious Vivaspin 2 with 200 nm pore size (Cat. #VS0271), and the second filtrate incorporated to the S-EVs fraction. Next, this combined S-EVs fraction was further ultra-filtered and concentrated using Sartorious Vivaspin 2 with 100 kDa pore size (Cat. #VS0241). Using this procedure, the original EVs obtained from HEI-OC1 cells were separated by size into two fractions, S-EVs (diameter ≤150 nm) and L-EVs (diameter >150 nm).

The counting and sizing of EVs in the L-EVs and S-EVs fractions was accomplished with the Microfluidic Resistive Pulse Sensing (MRPS^TM^) technique using a Spectradyne nCS1^TM^ instrument (Spectradyne LLC, Torrance, CA, USA; Cleland et al., 2016; Grabarek et al., 2019). This method is based on monitoring transient changes in electric current, also known as the Coulter principle, caused by particles passing through a narrow orifice (Song et al., 2017). When a nanoparticle passes through the constriction, it blocks some of the electrical sensing current, increasing the electrical resistance of the constriction by an amount proportional to the nanoparticle volume. Monitoring the electrical resistance as a function of time thus yields a number of short pulses, each corresponding to the passage of a nanoparticle, with the pulse amplitude yielding the nanoparticle volume, and the duration corresponding to the particle dwell time and thus the particle velocity. As the particles are entrained in the suspending fluid, the pulse duration yields the volumetric flow rate. One can thus obtain measurements of the concentration of nanoparticles as a function of nanoparticle size, directly from the temporal record of electrical resistance. This is a well-established technique, with the Coulter counter being the most commonly-used automated instrument for particle analysis (Vaclavek et al., 2019). At present, there are two commercially-available RPS instruments for counting and sizing EVs, the qNano (Izon Science Limited, New Zealand) and the nCS1^TM^ (Spectradyne LLC, Torrance, CA, USA; Vaclavek et al., 2019). The qNano instrument expands RPS technique to a broader range of particle diameters by using an elastic pore that can be stretched, an approach known as Tunable Resistive Pulse Sensing (TRPS; Weatherall and Willmott, 2015). This instrument, however, requires user-dependent manual settings that reduce data reproducibility (Tkach et al., 2018). Rather than a single elastic pore, the MRPS^TM^ technique in the nCS1^TM^ instrument uses disposable polydimethylsiloxane cartridges, each with a constriction of a particular size in a microfluidic channel acting as a sensing gate (Cleland et al., 2016).

**Figure 1:**
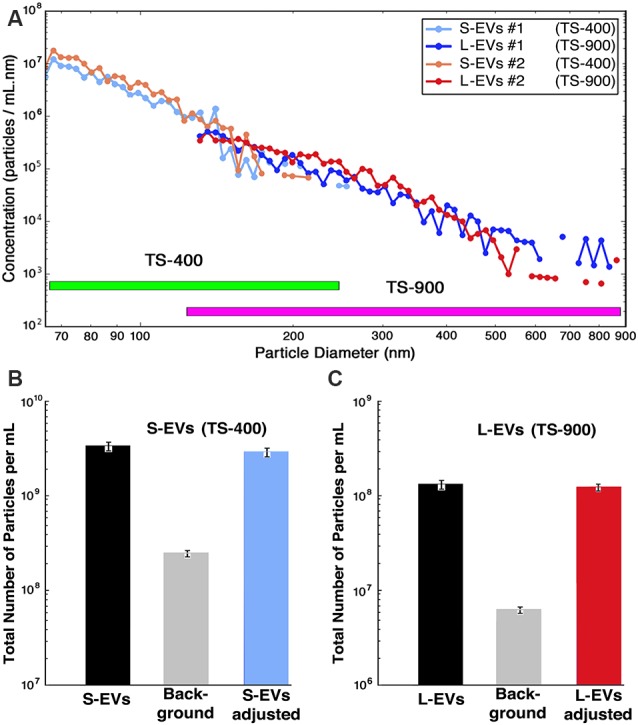
Counting and sizing HEI-OC1 extracellular vesicles (HEI-OC1 EVs). **(A)** Computer-generated concentration spectral density (CSD) vs. particle size for two independent samples (S1 and S2) in the whole size range. Values for S1 and S2 are already corrected by bin-by-bin background subtraction of the values obtained from matched CM+ED-FBS and PBS+0.1% BSA (see Supplementary Figure S1). Note that the TS-400 and TS-900 cartridges have an overlapping region. **(B)** Bar graphic depicting the total number of particles in the small-EVs (S-EVs) fraction [average of S1 and S2 = (3.3 ± 0.1) × 10^9^ particles/ml], the total background [(1.30 ± 0.04) × 10^8^ particles/ml for CM+ED-FBS plus (1.35 ± 0.08) × 10^8^ particles/ml for PBS+0.1% BSA = (2.65 ± 0.12) × 10^8^ particles/ml], and average of adjusted values [(3.3 ± 0.1) × 10^9^ – (2.65 ± 0.12) × 10^8^ = (3.0 ± 0.2) × 10^9^ particles/ml]. **(C)** Bar graphs depicting the total number of particles in the large-EVs (L-EVs) fraction [average of S1 and S2 = (1.39 ± 0.07) × 10^8^ particles/ml], the total background [(4.8 ± 0.2) × 10^5^ particles/ml for CM+ED-FBS plus (5.3 ± 0.4) × 10^6^ particles/ml for PBS+0.1% BSA = (5.8 ± 0.6) × 10^6^ particles/ml], and average of adjusted values [(1.39 ± 0.69) × 10^8^ – (5.8 ± 0.6) × 10^6^ = (1.3 ± 0.7) × 10^8^ particles/ml]. Note that in all panels the scale of the Y-axis is logarithmic, but panel **(A)** is in CSD units (particles/ml.nm) whereas panels **(B,C)** are in absolute concentration units (particles/ml).

In the present study, we used two different disposable polydimethylsiloxane cartridges, TS-400 and TS-900. TS-400 cartridges were utilized in experiments with S-EVs (range 65–400 nm particle size) and TS-900 cartridges in those with L-EVs (range 130–900 nm particle size; Cleland et al., 2016; Grabarek et al., 2019). Samples were first diluted with PBS (1:10) and then supplemented with 0.1% BSA as recommended by the instrument manufacturer, and counting and sizing determined as the average of triplicate measurements on two independent samples. Since the instrument does not discriminate between EVs generated by HEI-OC1 cells from those contributed by the FBS used in cell culture or the abundant silicate particles present in BSA solutions, the values for the number of particles per diameter were background-corrected by subtracting bin-by-bin the numbers obtained in matched control samples of both culture media containing exosome-depleted FBS (CM+ED-FBS) and PBS+0.1% BSA. Particle concentration is depicted in the figures provided by the instrument in the form of Concentration Spectral Density (CSD), which corresponds to the density of particles per ml of solution per nm of particle diameter (particles/ml.nm). The use of CSD allows the comparison of measurements made with different cartridges since the CSD is independent of the cartridge parameters and bin widths. The data, however, can be exported in either CSD or in absolute concentration (particles/ml), which is obtained by multiplying the CSD values by the width of the respective bins. In the present study, Figure 1A; Supplementary Figure S1 display CSD plots, but Figures 1B,C as well as numerical values in the text are provided in absolute concentration.

### Proteomic Studies

Bottom-up proteomics was performed using well-established protocols as recently described (Kalinec et al., 2019). Briefly, peptide samples were desalted using a modified version of Rappsilber’s protocol (Rappsilber et al., 2007) and fractionated *via* high pH reverse phase chromatography (Agilent Poroshell 120). The fractions were then analyzed by in-line nanobore reversed-phase chromatography coupled to nanospray ionization on a hybrid quadrupole-Orbitrap mass spectrometer (nLC-MS/MS; QE-Plus, Thermo Fisher Scientific, Waltham, MA, USA; Capri and Whitelegge, 2017). The data were processed using Proteome Discoverer 2.2 (Thermo Fisher Scientific, Waltham, MA, USA), which provides measurements of relative abundance for the identified peptides, and mined using mouse protein databases (Kanehisa and Goto, 2000; Kanehisa et al., 2014).

### Loading of EVs and Targeted Lipidomic Analysis

HEI-OC1 EVs were loaded with 10 mM aspirin (ASP; Catalog No. A5376, Sigma-Aldrich, St. Louis, MO, USA), arachidonic (AA), eicosapentaenoic (EPA), docosahexaenoic (DHA), and linoleic (LA) acids (Sigma-Aldrich, St. Louis, MO, USA), lipoxin A4 (LXA_4_) and resolvin D1 (RvD1; CAS No. 89663-86-5 and CAS No. 872993-05-0, respectively, Cayman Chemical, Ann Arbor, MI, USA), alone or combined. Loading was performed by co-incubation for 1 h at 25°C with sonication for 5 min (Kalinec et al., 2019), a procedure favored by the hydrophobic nature of the molecules selected as cargo (Armstrong et al., 2017). These particular molecules were chosen because they are either pro-resolving mediators (ASP, LXA4, RvD1) or precursors of pro-resolving mediators (AA, EPA, DHA, and LA). Unloaded drug was removed by tangential flow filtration followed by ultrafiltration and concentration as already described in “EVs Isolation and Physical Characterization” section. This procedure increases more than 20-fold the PBS washing volume, rendering a more thorough wash-out of any unincorporated drug than the second purification step with exoEasy^TM^ columns performed in our previous study (Kalinec et al., 2019).

For confirming ASP incorporation by S-EVs and L-EVs, dried samples were treated with *N*,*O*-bis(trimethylsilyl)trifluoroacetamide reagent (Pierce, 50 μl of reagent, 60°C, 60 min), and aliquots (1 μl) of the solution were analyzed by GC/MS (Thermo Q Exactive GC) for detection of the trimethylsilyl ASP derivative. Peak areas from the reconstructed ion chromatograms (m/z 195.04622-195.04818) for the fragment ion corresponding to the loss of a methyl and acetyl group from the molecular ion (observed m/z 195.04713, calculated 195.04720 for C_9_H_11_O_3_Si) were compared to those obtained from known amounts of ASP treated in the same way.

For targeted lipidomics, S-EVs and L-EVs were divided into 10 groups: S-Control: S-EVs untreated; S-EVs #2: S-EVs incubated with 10 mM ASP; S-EVs #3: S-EVs incubated with 10 mM LXA4 + 10 mM RvD1; S-EVs #4: S-EVs incubated with 10 mM AA+EPA+DHA+LA; S-EVs #5: S-EVs incubated with 10 mM AA+EPA+DHA+LA+ASP; L-Control: L-EVs untreated; L-EVs #2: L-EVs incubated with 10 mM ASP; L-EVs #3: L-EVs incubated with 10 mM LXA4+RvD1; L-EVs #4: L-EVs incubated with 10 mM AA+EPA+DHA+LA; L-EVs #5: L-EVs incubated with 10 mM AA+EPA+DHA+LA+ASP. Samples of each of these groups were sent to the UC San Diego Lipidomic Core for fatty acid and eicosanoid analysis^1^. As previously described (Eguchi et al., 2016), samples were supplemented with 26 deuterated internal standards and brought to a volume of 1 ml with PBS containing 10% methanol. They were then partially purified by solid-phase extraction on Strata-X columns (Phenomenex, Torrance, CA, USA) following the procedure outlined by the manufacturer. The columns were eluted with methanol (1 ml), the eluent was dried under vacuum and redissolved in 50 μl of buffer A (water/acetonitrile/acetic acid, 60:40:0.02 (v/v/v) and immediately used for LC-MS analysis. Eicosanoids were analyzed as previously described (Quehenberger et al., 2010, 2011). Briefly, eicosanoids were separated by reverse-phase chromatography using a 1.7-μM 2.1 × 100-mm BEH Shield Column (Waters, Milford, MA, USA) and an Acquity UPLC system (Waters, Milford, MA, USA). The column was equilibrated with buffer A and 5 μl of sample was injected *via* the autosampler. Samples were eluted with a step gradient to 100% buffer B consisting of acetonitrile/isopropanol = 50:50 (v/v). The liquid chromatography effluent was interfaced with a mass spectrometer, and mass spectral analysis was performed on an AB SCIEX 6500 QTrap mass spectrometer equipped with an IonDrive Turbo V source (AB SCIEX, Framingham, MA, USA). Eicosanoids and polyunsaturated fatty acids (PUFA) were measured using multiple reaction monitoring (MRM) transitions with the instrument operating in the negative ion mode (Wang et al., 2014). Collisional activation of the eicosanoid precursor ions was achieved with nitrogen as the collision gas, and eicosanoids were identified by matching their MRM signals and chromatographic retention times with those of pure identical standards. Detailed instrument settings are summarized elsewhere (Quehenberger et al., 2018). Eicosanoids were quantified by the stable isotope dilution method. Briefly, identical amounts of deuterated internal standards were added to each sample and to all the primary standards used to generate standard curves. To calculate the amounts of eicosanoids in a sample, ratios of peak areas between endogenous eicosanoids and matching deuterated internal eicosanoids were calculated. Ratios were converted to absolute amounts by linear regression analysis of standard curves generated under identical conditions.

## Results

### Counting and Sizing EVs

Previous studies of our laboratory showed that auditory HEI-OC1 cells generate abundant EVs (Kalinec et al., 2019). This earlier study, however, suggested that they were mostly L-EVs, with a significant lower number of S-EVs. This was an unexpected result that, if confirmed, would indicate that the mechanism of EV generation could be different in HEI-OC1 than in other cell populations. Thus, the first goal of the present study was to evaluate the number of S-EVs and L-EVs generated by HEI-OC1 cells. Since the nanotracking technique used in our previous study is dependent on the optical properties of the particles, which vary with their size, we switched to a different technique, Microfluidic Resistive Pulse Sensing (MRPS).

The data provided by the nCS1^TM^ MRPS instrument, using TS-400 and TS-900 cartridges, is summarized in Figure 1. The number of particles vs. diameter of particle curves corresponding to S-EVs and L-EVs from two independent samples (#1 and #2) are depicted in Figure 1A, with values expressed in CSD units (particles/ml.nm). Each point of these curves represents the average of three measurements per sample, and they are already adjusted by bin-by-bin background subtraction of the values obtained from matched CM+ED-FBS and PBS+0.1% BSA control samples (confidence intervals are depicted by the diameter of the graphic points corresponding to every value; for background values, also in CSD units, see Supplementary Figure S1). The data shows that the number of EVs varies in inverse proportion to their size, with a maximum of around 1 × 10^7^ particles/ml and a minimum of about 1 × 10^5^ particles/ml for S-EVs, and a range of around 1 × 10^6^ to 1 × 10^3^ particles/ml for L-EVs (note that these values are expressed in absolute concentration units). When the areas under the size distribution plots were summed, and background subtracted, the S-EVs samples contained an adjusted average of (3.0 ± 0.2) × 10^9^ particles/ml, corresponding to a total of (3.3 ± 0.1) × 10^9^ particles/ml minus (2.65 ± 0.08) × 10^8^ particles/ml for background (Figure 1B). L-EVs samples, in turn, contained an adjusted average of (1.3 ± 0.7) × 10^8^ particles/ml, as a consequence of a total value of (1.39 ± 0.69) × 10^8^ particles/ml in experimental samples and (5.8 ± 0.4) × 10^6^ particles/ml for background (Figure 1C). Thus, in contrast to the results using the nanotracking technique (Kalinec et al., 2019), our present data indicate that HEI-OC1 cells generate more S-EVs than L-EVs. Other information apparent from these figures is that the fractions are not absolutely “pure,” since the S-EVs one still contains a certain number of particles with diameters larger than 150 nm and, vice-versa, the L-EVs fraction contains vesicles with diameters smaller than 150 nm (Figure 1A).

Interestingly, using the formulas *V* = 4/3 (πr^3^) and *S* = 4 πr^2^, the particle diameters provided by the nCS1^TM^ instrument to estimate the EVs’ radius, and assuming the EVs are perfectly spherical, the calculated total volumes (S-EVs = 3.1 × 10^14^ nm^3^; L-EVs = 6.4 × 10^14^ nm^3^) and surface (membrane) areas (S-EVs = 1.8 × 10^13^ nm^2^; L-EVs = 1.2 × 10^13^ nm^2^) of the vesicles present in each fraction are of the same order of magnitude.

### Protein Profiling of HEI-OC1 EVs

Next, we wonder whether S-EVs and L-EVs shared a similar protein profile or, instead, they contain some unique protein markers that could be used for identification or classification. To investigate this issue, we performed bottom-up proteomics.

A total of 620 *bona fide* EV proteins were detected in our proteomic studies, 489 of them in the S-EVs fraction and 131 in the L-EVs fraction (Supplementary Tables S1, S2). From them, 381 (61.5%) were unique for S-EVs, 23 (3.7%) were specific for the L-EVs, and 108 (17.4%) were found in both fractions. Interestingly, 86.7% (26 out of 30) of the proteins listed in the ExoCarta exosome database as those more frequently identified in exosomes (Keerthikumar et al., 2016) were detected in S-EVs; in contrast, only 15 out of these 30 (50%) were detected in the L-EVs fraction. CD63, CD81, and PDCD6IP (*aka* Alix), all considered exosome biomarkers, were identified only in S-EVs (Table 1). More importantly, the protein mediators of inflammatory resolution Annexin A1 (ANXA1) and Galectins 1 and 3 (Gal-1 and Gal-3) were only detected in the S-EVs fraction too (Supplementary Tables S1, S2). On the other hand, only one cytokine (CSF1, colony-stimulating factor 1) was detected in HEI-OC1 EVs, but it was present in both fractions S-EVs and L-EVs.

**Table 1:** Expression in small extracellular vesicles (S-EVs) and large EVs (L-EVs) fractions of the proteins more frequently identified in exosomes.

Score ExoCarta	Gene symbol	Present in S-EVs?	Present in L-EVs?
1	CD9	-	-
2	HSPA8	YES	YES
3	PDCD6IP	YES	-
4	GAPDH	YES	YES
5	ACTB	YES	YES
6	ANXA2	YES	-
7	CD63	YES	-
8	SDCBP	-	-
9	ENO1	YES	YES
10	HSP90AA1	YES	YES
11	TSG101	-	-
12	PKM	YES	YES
13	LDHA	YES	-
14	EEF1A1	YES	YES
15	YWHAZ	YES	YES
16	PGK1	YES	YES
17	EEF2	YES	YES
18	ALDOA	YES	-
19	HSP90AB1	YES	YES
20	ANXA5	YES	-
21	FASN	-	-
22	YWHAE	YES	YES
23	CLTC	YES	-
24	CD81	YES	-
25	ALB	YES	YES
26	VCP	YES	YES
27	TPI1	YES	-
28	PPIA	YES	-
29	MSN	YES	-
30	CFL1	YES	YES

*ExoCarta: http://exocarta.org/Archive/ExoCarta_top100_protein_details_5.txt. Accessed January 29, 2019. Twenty-six out of the 30 (86.7%) proteins more frequently identified in exosomes were found in S-EVs, but only 15 (50%) of them were found in L-EVs*.

**Figure 2:**
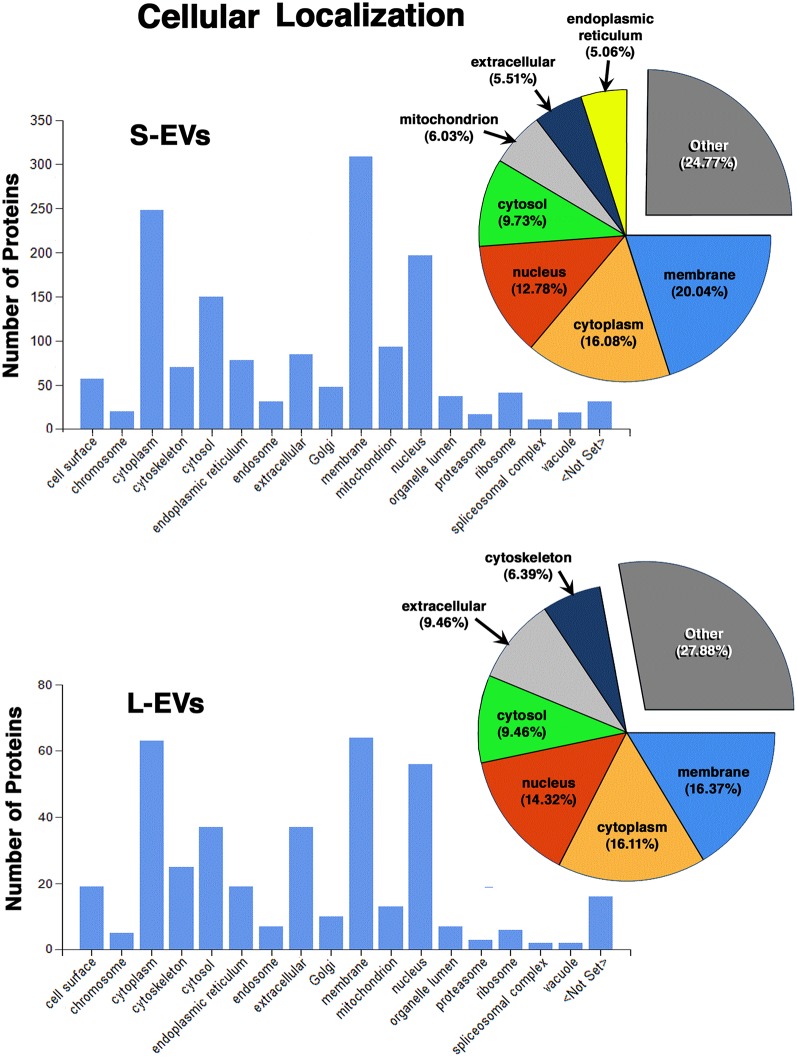
Proteomic analysis. Characterization of proteins from HEI-OC1 EVs by cellular localization.

**Figure 3:**
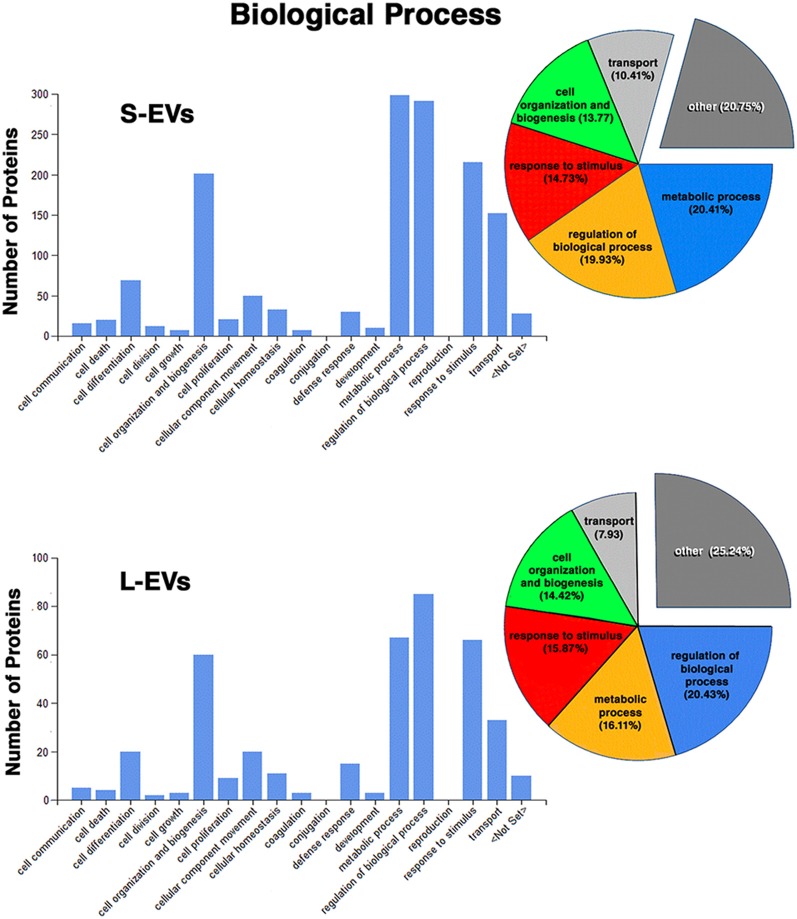
Proteomic analysis. Characterization of proteins from HEI-OC1 EVs by associated biological process.

**Figure 4:**
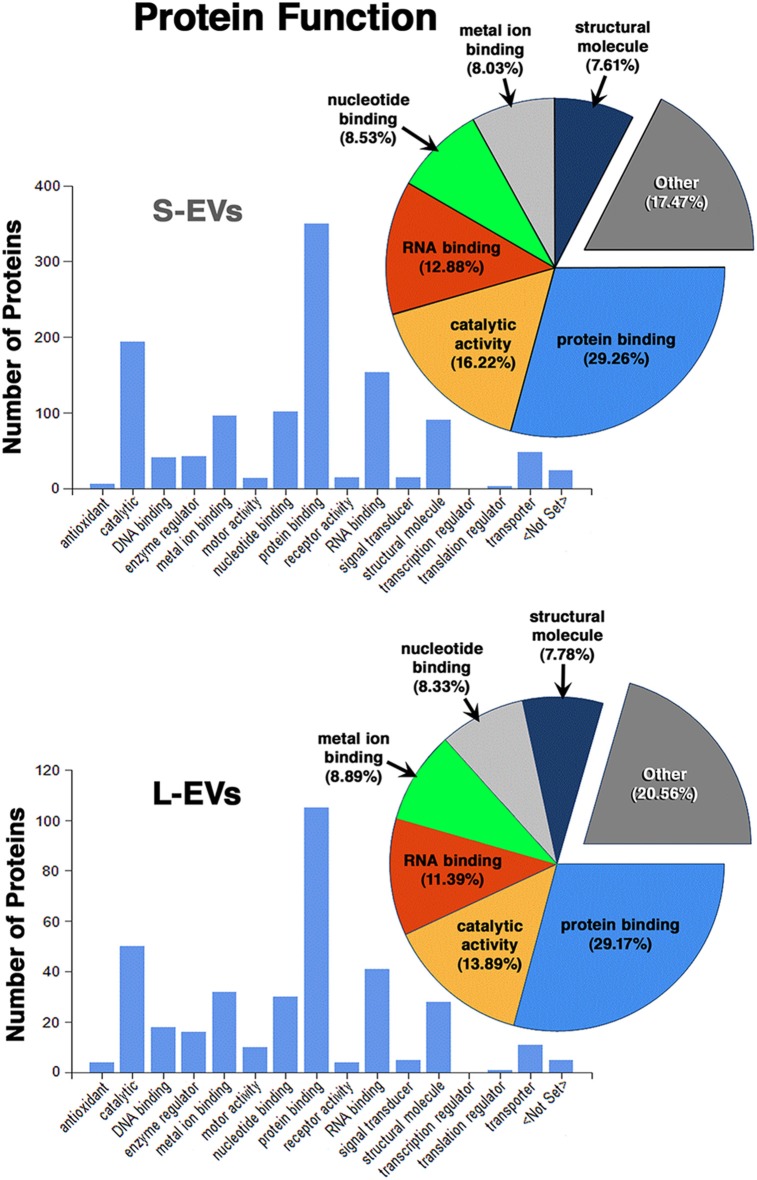
Proteomic analysis. Characterization of proteins from HEI-OC1 EVs by molecular function.

Except for the difference in the total number of proteins in each fraction, the distribution profiles by cellular origin, biological and molecular functions were quite similar in S- EVs and L-EVs (Figures 2–4). Most of the proteins detected in both fractions originated from cell membranes, cytoplasm, nucleus, or cytosol, with minor percentages of extracellular origin or from organoids (mitochondria, endoplasmic reticulum) and the cytoskeleton (Figure 2). They were mostly involved in metabolism or regulation of other biological processes, as well as in response to stimuli, cell organization and biogenesis, and transport (Figure 3). The most common function was molecular binding, either to other proteins, RNA, metal ions or nucleotides, but proteins with catalytic or structural function were also significantly represented (Figure 4). In every case, the proteins identified as “Others” include all those belonging to groups with less than 5% of the total.

In summary, we found that the S-EVs fraction contains a larger number and greater diversity of proteins than the L-EVs, but the similarities observed in their distribution profiles (Figures 2–4) suggest that this difference could be associated with the involvement of a more efficient cellular mechanism of protein sorting and/or loading into S-EVs from a single pool of molecules rather than the existence of two different pools, one for S-EVs and other for L-EVs. The presence of ANXA1, Gal-1, and Gal-3 makes S-EVs more attractive as potential nanocarriers in pro-resolving therapies.

### EVs’ Loading and Target Lipidomic Analysis

The absence of toxic effects and the ability to incorporate as cargo the drugs and molecules of interest are crucial requirements for a useful drug carrier. As already mentioned, EVs are not toxic, lack endogenous tumor-formation potential, and they show very low immunogenicity. In addition, they can be easily loaded with pharmacological agents using simple procedures. Therefore, we decided to investigate the loading of S-EVs and L-EVs from HEI-OC1 cells with ASP, the eicosanoids LXA4, RvD1, and the PUFA AA, DHA, EPA, and LA, all of them recognized anti-inflammatory and pro-resolving agents. While ASP incorporation was evaluated by GC/MS, eicosanoids were identified and quantified by targeted lipidomics using LC/MS/MS-MRM. Importantly, in addition to revealing the identity and concentration of around 150 eicosanoids, PUFA, and related compounds, the lipidomic profiles revealed the presence and amounts of endogenous pro-inflammatory components that could counter the pro-resolving effects of the cargo.

Co-incubation of HEI-OC1 EVs (10 ml, with 1 × 10^8^ S-EVs and L-EVs per ml) with ASP (10 mM) resulted in the incorporation of 6.9 ± 0.1 μg/ml (~38 μM) of ASP in S-EVs samples, and 61.8 ± 0.6 μg/ml (~0.34 mM) ASP in L-EVs samples. These results suggest that EVs, particularly L-EVs could be loaded with pharmacologically effective quantities of this drug.

**Figure 5:**
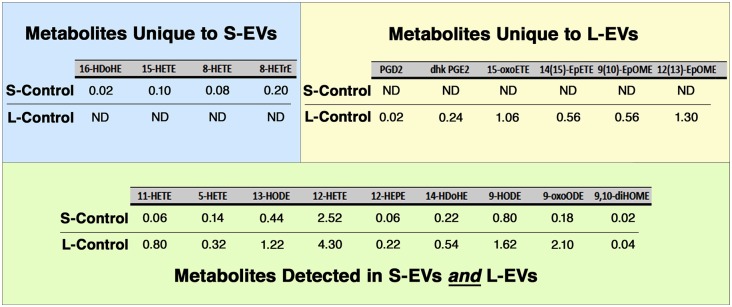
Lipidomic analysis. Only 19 out of 150 metabolites investigated were detected in Control HEI-OC1 EVs, four of them in S-EVs, six in L-EVs, and nine in both fractions. Values expressed in pmol/ml. ND, Not Detected.

Of the 150 components of the lipidomic profiles, only 19 were detected in untreated (Control) samples, with four of them detected only in S-Control, six only in L-Control, and nine found in both fractions (Figure 5, Supplementary Table S3). In addition, while free AA, DHA, and EPA were found in untreated EVs, LXA4, ATL-LXA4, Resolvins, Maresins, and Protectins were not detected (Table 2, Supplementary Table S3).

**Table 2:** Lipidomic analysis.

Group	Free AA	Free EPA	Free DHA	AT-LXA_4_	LXA_4_	RvD1
S-Control	17.84	0.52	6.02	ND	ND	ND
S-EVs #2	28.86	0.72	10.34	ND	ND	ND
S-EVs #3	50.02	2.20	19.62	1.56	55.40	15.44
S-EVs #4	6,370.88	4,764.42	5,149.18	20.26	24.46	ND
S-EVs #5	5,872.28	4,633.08	5,625.40	13.36	15.94	ND
L-Control	154.54	94.24	110.38	ND	ND	ND
L-EVs #2	73.98	38.58	47.52	ND	ND	ND
L-EVs #3	25.28	12.64	13.92	6.26	207.34	70.70
L-EVs #4	3,596.42	2,707.80	3,438.06	8.00	9.14	ND
L-EVs #5	2,183.12	2,387.16	2,393.28	7.36	9.24	ND

*Incorporation of fatty acids and pro-resolving mediators by HEI-OC1 EVs. S-Control: S-EVs untreated; S-EVs #2: S-EVs incubated with 10 mM ASP; S-EVs #3: S-EVs incubated with 10 mM LXA4 + 10 mM RvD1; S-EVs #4: S-EVs incubated with 10 mM AA+EPA+DHA+LA; S-EVs #5: S-EVs incubated with 10 mM AA+EPA+DHA+LA+ASP; L-Control: L-EVs untreated; L-EVs #2: L-EVs incubated with 10 mM ASP; L-EVs #3: L-EVs incubated with 10 mM LXA4+RvD1; L-EVs #4: L-EVs incubated with 10 mM AA+EPA+DHA+LA; L-EVs #5: L-EVs incubated with 10 mM AA+EPA+DHA+LA+ASP. Results are expressed in pmol/ml. ND, Not Detected*.

As shown in Table 2 both, S-EVs #3 and #4 and L-EVs #3 and #4, were able to simultaneously incorporate AA, EPA, and DHA in amounts between 5 and 6 nmol per ml of suspension (about 1.5–2.0 ng/ml). Importantly, in EVs loaded with these PUFA, all 19 metabolites originally detected in S-Control and L-Control samples (Figure 5) showed an increased concentration in both fractions. Moreover, 50 other metabolites previously undetected were found in these EVs, making a total of 69 eicosanoids identified in the samples (Supplementary Table S3). Inflammatory agents were not detected in Control and ASP-loaded EVs, but those loaded with PUFAs generate several prostaglandins [e.g., PGD_2_(189.18 pmol/ml], PGE_2_(48.94 pmol/ml), PGF_2a_ (15.64 pmol/ml), leukotriene B4 (LTB_4_, 3.84 pmol/ml), and thromboxane B2 (TXB2, 1.18 pmol/ml), among others (Supplementary Table S3). The generation of PUFA derivatives suggests the existence of an active biosynthetic mechanism within HEI-OC1 EVs. LXA4 and AT-LXA4 (ASP triggered-LXA4) were also detected in these groups in amounts varying roughly between 3 and 10 pmol/ml (about 0.35 ng/ml). Since the limit of detection of the lipidomic experiments was around 0.002 pmol/ml, the measured values represent increases of at least 3–4 orders of magnitude (1,000-fold to 10,000-fold) relative to the control conditions. As expected, S-EVs and L-EVs also incorporated LXA4 and RvD1, although in lower amounts than their precursors AA, EPA, and DHA. It should be noted, however, that the amounts of LXA4 and RvD1 incorporated by HEI-OC1 EVs ranged from 15 to 210 pmol/ml (roughly 5–70 μg/ml), six orders of magnitude higher than the normal concentrations of these pro-resolving mediators in human serum [2–120 pg/ml (Colas et al., 2014; Dalli et al., 2017)]. Curiously, AT-LXA4 was not found in ASP-loaded EVs (S-EVs #2 and #5, and L-EVs #2, and #5), but was detected in EVs loaded with LXA4 (S-EVs #3 and L-EVs#3) and AA, its fatty acid precursor (S-EVs #4 and #5, and L-EVs #4, and #5).

## Discussion

The present study provides evidence that auditory HEI-OC1 cells generate abundant EVs, both small (<150 nm diameter) and larger (>150 nm diameter), which can be loaded with anti-inflammatory drugs, PUFA, and pro-resolving mediators, either alone or combined, in amounts significantly higher than those normally found in human serum. Proteomic studies detected a differential presence of selected proteins in S-EVs and L-EVs fractions. In particular, S-EVs contained a larger variety of proteins, including the pro-resolving mediators ANXA1, Gal-1 and Gal-3, than L-EVs. Targeted lipidomic studies, in turn, identified eicosanoids that were present in one of the EV fractions and not in the other. These results support the idea that HEI-OC1 EVs could be advantageously used as nanocarriers for delivery of drugs and molecular mediators aimed at facilitating the resolution of inflammatory processes. In particular, they could potentially be useful as vehicles for the intracochlear delivery of pro-resolving agents aimed at preventing or alleviating DRHL and NRHL.

### Counting and Sizing EVs

The present results indicate that HEI-OC1 cells secrete near two orders of magnitude more S-EVs than L-EVs, with the number of EVs decreasing monotonically with their increase in diameter (Figure 1A). Since the instrument used for the measurements counts individual particles and estimates their diameter, arithmetic calculations from the data (assuming that all the EVs were perfect spheres) indicated that the total volume and total membrane area of the vesicles present in each fraction were of the same order of magnitude, suggesting that the drug-loading capability of both fractions should be roughly similar.

The abundance of S-EVs is in contradiction to the data we reported in a recent publication, where the counting and sizing of HEI-OC1 EVs were based on light-scattering technology (Kalinec et al., 2019). The distribution of EVs by size as estimated with that technique showed a low concentration of particles with diameters below 200 nm, suggesting that HEI-OC1 EVs consisted mostly of L-EVs. However, as already discussed in that publication, although currently considered as reliable, light-scattering techniques are not exempt from problems (Witwer et al., 2013; Koritzinsky et al., 2017; Grabarek et al., 2019). In particular, particles with an optical index close to that of the suspension medium or with high curvature radius (small diameter) scatter much less light than those with a significantly different index or larger diameter. This sensitivity dependence makes small biological particles essentially undetectable (Cleland et al., 2016; Grabarek et al., 2019), a fact that could explain the difference between our preliminary results and those reported here.

In the present study, HEI-OC1 EVs were counted and sized with an instrument (Spectradyne nCS1^TM^) based on a completely different technique, Microfluidic Resistive Pulse Sensing (MRPS^TM^; Cleland et al., 2016; Grabarek et al., 2019). MRPS is based on monitoring transient changes in electric current, also known as the Coulter principle, caused by particles passing through a narrow orifice (Song et al., 2017). Using the nCS1^TM^ for counting and sizing, EVs have some important advantages. The nCS1^TM^ is capable of measuring the size distribution of EVs with diameters ranging from 35 nm up to 10 μm over concentrations ranging from 10^7^ to 10^12^ particles/ml, and statistically significant data sets can be acquired rapidly (minutes). Because the nCS1^TM^ uses electrical sensing, not optical detection, measurements are independent of the material properties of the particles. Importantly, the small sample volume required for analysis (3 μl) is set by the size of the analyte reservoir in the disposable cartridge. Moreover, the measurement does not rely on user-adjustable parameters, rendering more reproducible results. Although not completely free of limitations (analysis of particles with diameters <55 nm with this technique is still complicated, and the use of different cartridges makes cumbersome the evaluation of samples with a big range of particle’s diameters), MRPS is probably the best currently available technique for analyzing particles in the nanometer range (Grabarek et al., 2019).

### Protein Profiles of S-EVs and L-EVs

A recent “consensus” publication, co-authored by several prominent scientists in the field of EVs, stated that “EVs in the small size range likely represent vesicles heterogeneous in origin” with unknown portions of exosomes and ectosomes (Mateescu et al., 2017). The definition of larger EVs is even less precise, with these vesicles comprising a wide range of membrane-enclosed entities. Indeed, it is not yet clear how to divide EVs into their relevant subtypes, or even how many functionally distinct subtypes are there (Mateescu et al., 2017). In addition, every EV subtype could have different protein and lipid composition and, consequently, a different function (Kowal et al., 2016; Willms et al., 2016). Thus, recent studies have searched for a better characterization of different EV subtypes, and one of the common classifications is division by size into small and large (Vagner et al., 2019). A similar approach was followed in the present study.

Our results show that S-EVs and L-EVs do not contain the same repertoire of proteins. In fact, only 17.4% (108 out of the 620) proteins identified as belonging to HEI-OC1 EVs are common to both fractions, whereas 61.5% (381 out of 620) are unique to S-EVs and 3.7% (23 out of 620) were found only in L-EVs. As shown in Table 1, 86.7% of the proteins more frequently found in exosomes were present in S-EVs from HEI-OC1 cells, whereas only 50% were also present in the L-EVs fraction. Two proteins (SDCBP, an adapter protein involved in exosome biogenesis, and FASN, a fatty acid synthase) frequently identified in exosomes and previously reported in HEI-OC1 EVs (Kalinec et al., 2019) were not detected in the present study. Interestingly, the protein most frequently found in exosomes from other cell populations, tetraspanin CD9, was also absent in EVs from HEI-OC1 cells. This confirmed our preliminary results (Kalinec et al., 2019), and those of others, suggesting that not all S-EVs have the same tetraspanin profile (Vagner et al., 2019). For instance, CD9 and CD63 were variably detected in S-EVs and L-EVs, whereas CD81 was exclusively detected in S-EVs. However, only the co-localization of CD81 with CD63 qualifies an S-EV as an exosome (Kowal et al., 2016; Tkach et al., 2018). Thus, we can conclude that our S-EVs samples probably contain classical exosomes since CD63 and CD81 were abundant in this fraction (Table 1).

Other difference between our current and earlier preliminary results was the finding of ANXA1 and ANXA5 in S-EVs (Supplementary Tables S1, S2). We commented in our previous work the oddity that “two of the most common annexins found in exosomes (ANXA1 and ANXA5) were either found at low levels or not appeared at all” in our samples (Kalinec et al., 2019). The fact that both of them were now found only in the S-EVs fraction suggests that they were probably present in our preliminary samples, but in non-detectable levels. The presence of ANXA1, in particular, provides important support to our idea of using HEI-OC1 EVs as nanocarriers of drugs and molecular mediators aimed at the resolution of inflammatory responses in the cochlea. ANXA1 (Annexin A1) is a potent anti-inflammatory and pro-resolving protein (see Kalinec et al., 2017 and references therein). Many of the cellular and molecular processes associated with the anti-inflammatory properties of glucocorticoids are modulated by ANXA1, and it is considered an important modulator of both the innate and adaptive immune systems.

Interestingly, the authors of a recent publication (Jeppesen et al., 2019) proposed that ANXA1 would be a specific marker of microvesicles shed from the plasma membrane, and ANXA5 a component of apoptotic vesicles. The subcellular distribution of ANXA1 is unusual (for instance see Buckingham and Flower, 2017); it is abundant in the cytoplasm of some cell populations, but a small proportion is also found on the external surface of the plasma membrane or attached to their inner leaflet. Sometimes, even a single membrane pool of ANXA1 has been detected in particular cells. In guinea pig Hensen cells (Kalinec et al., 2009) and HEI-OC1 cells, ANXA1 was found in the cytoplasm, with membrane localization observed only after stimulation with glucocorticoids; in contrast, Jeppesen et al. (2019) localized ANXA1 only in the plasma membrane of DKO-1 and Gli36 human cancer cells, which they used in the reported study. Therefore, while Jeppesen et al. (2019) detected only membrane-bound ANXA1 in microvesicles from human cancer cells, cytoplasmic ANXA1 could be abundant in exosomes from auditory HEI-OC1 cells. Likewise, although ANXA5 is prominent in apoptotic vesicles, its presence in exosomes or microvesicles from some cell populations cannot be discarded.

We also confirmed the presence in S-EVs of Gal-1 and Gal-3 (Supplementary Tables S1, S2), a family of glycan-binding proteins that regulate the initiation, amplification and resolution of acute and chronic inflammatory responses (see Kalinec et al., 2017 and references therein). Gal-1 has been associated with a range of anti-inflammatory effects on various cell types, whereas Gal-3 enhances the phagocytic capabilities of neutrophils, a property that may in part account for its protective role in infections. On the other hand, the only pro-inflammatory cytokine detected in HEI-OC1 EVs, the Colony Stimulating Factor 1 (CSF-1), was found in both fractions. CSF-1 is known to be involved in the proliferation, differentiation, and survival of monocytes and macrophages, and it has been implicated in promoting tissue repair following injury (Zhang et al., 2012). Recently, however, it was proposed that CSF-1 has a dual role as pro-inflammatory and anti-inflammatory/regulatory cytokine dependent on the particular immune response (Bhattacharya et al., 2015).

### Lipids in S-EVs and L-EVs

Lipids are involved in a number of crucial cellular functions. For instance, in addition to be essential components of cellular membranes, lipid mediators play important roles in cell signaling, including inflammation and immunity (Poorani et al., 2016). It is well known that specific lipids are enriched in EVs compared to their parent cells (Skotland et al., 2017), and there are several possible explanations for these dissimilarities. The composition of the cell medium is important for the lipid composition of the cells and most probably for EVs lipid profile too (Stoll and Spector, 1984). Different growth conditions may also contribute, since different cell densities may change the lipid composition of a cell and intracellular trafficking pathways (Kavaliauskiene et al., 2014). However, since in our experiments all these factors were identical for S-EVs and L-EVs, any difference should be associated with the precise origin of the particles.

LXA4, AT-LXA4, Resolvins, Maresins, and Protectins, in contrast to free AA, DHA, and EPA, were not detected in S-Control and L-Control (Table 2, Supplementary Table S3). On the other hand, four eicosanoids unique to S-EVs and six unique to L-EVs were detected in Control samples, while another nine were common to both fractions (Figure 5). Those unique to S-Control were the HydroxyEicosaTetraEnoic acids 8-HETE, 15-HETE, and 8-HETrE, which derive from AA, and 16-HDoHE (HydroxyDocosaHexaEnoic acid), an autoxidation product of DHA (Poorani et al., 2016; for a complete list of eicosanoid abbreviations see Supplementary Table S3, worksheet 3). These products of PUFAs are pre-dominantly pro-inflammatory, but they are also intermediaries in the generation of lipoxins and epi-lipoxins and enhance the generation of nitric oxide (NO), another pro-resolving mediator (Kalinec et al., 2017). Interestingly, two pro-inflammatory eicosanoids, prostaglandin D2 (PGD2) and 13,14-dihydro-15-keto-PGE2 (DHK-PGE2), were detected in L-Control but not in S-Control (Figure 5). In mammals, large amounts of PGD2 are found only in mast cells and the brain; in fact, PGD2 is the most abundant prostaglandin in the brain and the one that changes the most under pathological conditions (Figueiredo-Pereira et al., 2015). While prostaglandin E2 (PGE2) and 15-deoxy-Δ12,14-prostaglandin J2 (15-d-PGJ2) are frequently found in EVs (Subra et al., 2010), to our knowledge the literature only reports the presence of PGD2 in EVs in one study performed on exosomes from RBL-2H3 cells, a mast cell model (Subra et al., 2010). Other eicosanoids only detected in L-Control were 15-oxoETE (oxo EicosaTetraEnoic acid, an oxylipin produced by oxidation of 15-HETE), 14(15)-EpETE [a cytochrome P450 (CYP)-metabolite of EPA], 9(10)-EpOME and 12(13)-EpOME (products of the metabolism of linoleic acid by CYP enzymes). Just like all those detected in both fractions, these metabolites participate in pro-inflammatory and pro-resolving pathways, suggesting they are part of a delicate physiological balance.

An important piece of information provided by our lipidomic studies was the significant increase in the variety and amount of lipid metabolites present in EVs loaded with AA, EPA, and DHA (see below). More than 50 metabolites undetected in S-Control or L-Control were found in these EVs, making a total of 69 eicosanoids identified in our samples (Supplementary Table S3). For instance, in the absence of exogenously added PUFAs few pro-inflammatory eicosanoids were detected, and those that were detected were present at trace levels. In contrast, prostaglandins PGD2, DHK-PGE2, PGF2α, PGE2, PGE1, PGD1, PGE3, PGD3, 15K PGF2α, 15K PGE2, DHK-PGD2, 11β PGE2, Thromboxane B2 (TXB2), and other pro-inflammatory agents were detected in both S-EVs and L-EVs loaded with PUFAs (Supplementary Table S3). The data suggest that all pathways, including those mediated by COX (cyclooxygenase), 5-LOX (lipooxygenase), 12-15 LOX and CYP (cytochrome P450), are endogenously present in the HEI-OC1 EVs, and are sufficiently active to produce abundant molecular products when supplied with substrates. It should be emphasized that, since EVs were loaded exogenously in the absence of cells, we can confidently assume that all these molecular products were generated in the EVs, suggesting the existence in EVs of an active metabolic mechanism.

Most of these AA, EPA and DHA metabolites are part of pathways that give rise not only to potent pro-resolving mediators but also to pro-inflammation agents. Many of the prostaglandins, including the highly induced PGD2, are considered pro-inflammatory. In general, under steady-state physiological conditions, one would expect a balance between pro- and anti-inflammatory molecules so that homeostasis is preserved. When this balance is altered, depending on the side to which it is tilted, either there will be suppression or exaggeration of inflammation. Our results suggest that incorporation of PUFAs as cargo induces the generation of pro-inflammatory agents in the HEI-OC1 EVs, which could interfere with the goal of accelerating the resolution of inflammatory processes. Thus, if HEI-OC1 EVs are used as nanocarriers, their cargo should be carefully tailored to ensure that it will promote the return to the normal balance.

### Loading of S-EVs and L-EVs

EVs are able to incorporate as cargo different types of molecules, such as miRNAs, messenger RNAs, siRNAs, proteins, and even drugs like doxorubicin and curcumin (Sun et al., 2010; Lai et al., 2013; Yang et al., 2015). Moreover, EVs can be loaded exogenously or endogenously. In the first approach, the molecule of interest is included in EVs after isolation, while the endogenous approach is based on active encapsulation, particularly during EV biogenesis, *via* transforming the producing cells with the selected molecule (Pinheiro et al., 2018). EVs loading, however, depends on different factors, including the cellular origin of the EVs, which dictates their molecular composition, and the physical-chemical properties of the intended cargo (e.g., hydrophobicity). In the case of hydrophilic drugs, for instance, special strategies such as hydrophobic insertion, covalent surface chemistry, and membrane permeabilization, may be necessary to facilitate loading (Armstrong et al., 2017). Incorporation of drugs like dexamethasone and aspirin inside EVs, on the other hand, should not be a surprise, since they are hydrophobic. Likewise, fatty acids and lipid mediators should be easily incorporated. However, the loading of other drugs and molecules of interest by HEI-OC1 EVs must be demonstrated before serious consideration of their use as nanocarriers.

Incorporation of dexamethasone by HEI-OC1 EVs was already reported in our preliminary study (Kalinec et al., 2019), and here we present proof that they can be loaded with ASP, LXA4, RvD1, and the eicosanoid precursors AA, EPA, and DHA. These results support the idea that HEI-OC1 EVs could be ideal vehicles for intracochlear delivery of drugs and molecular mediators aimed at facilitating the resolution of cochlear inflammatory processes.

The amount of ASP incorporated by HEI-OC1 EVs was surprisingly high, with significantly more loading by L-EVs (61.8 ± 0.6 μg/ml) than by S-EVs (6.9 ± 0.1 μg/ml). Since the volume of L-EVs per ml is only about twice the volume of S-EVs per ml (6.4 × 10^14^ nm^3^ vs. 3.1 × 10^14^ nm^3^), we speculate that could be some structural problem limiting ASP incorporation in S-EVs. For instance, the different loading of ASP by S-EVs and L-EVS could be related to differences in the curvature of the EV membrane and/or surface charge (Zhou and Raphael, 2007). It has been shown that ASP interacts with membrane lipids, being incorporated first to the external layer and translocating to the internal by flip-flop before being internalized. During this process, the physical properties of the full structure, including their thickness, bending elasticity, and permeability, are affected (Zhou and Raphael, 2005; Sharma et al., 2017). These changes could be more significant in small EVs, hindering the incorporation of ASP, while L-EVs could more readily accommodate the salicylate molecules (Zhou and Raphael, 2007).

ASP, in addition to its proven anti-inflammatory, pro-resolving, and anti-oxidant properties, is the only drug to date that has shown beneficial effects for the mitigation of sensorineural hearing loss in clinical trials (see Kalinec et al., 2017 and references therein). ASP not only blocks the biosynthesis of prostaglandins but also stimulates the endogenous production of pro-resolving mediators, such as ASP-triggered lipoxins (AT-LXs) and resolvins (AT-RVs), which promote the resolution of inflammation by stimulating phagocytosis of cellular debris and counter-regulate pro-inflammatory cytokines without being immunosuppressive (Serhan, 2014). ASP-triggered pro-resolving mediators are generated by the activity of ASP-acetylated cyclooxygenase on PUFA substrates, including AA, EPA, and DHA (Clària and Serhan, 1995; Clària et al., 1996; Serhan et al., 2002; Dalli et al., 2013; Serhan, 2014). In addition to exhibiting similar anti-inflammatory and pro-resolving characteristics of native mediators, the ASP-triggered forms (R epimers) resist rapid inactivation by oxido-reductases and have longer *in vivo* half-lives (Serhan, 2014). Intriguingly, our present results indicate that incorporation of ASP as cargo did not produce detectable amounts of AT-LXA4 in HEI-OC1 EVs in contrast to the significant amounts found in EVs loaded with AA, EPA and DHA (Table 2: compare groups S-EVs #2 and L-EVs #2 with S-EVs #4 and #5, and L-EVs #4 and #5). These results present a puzzle: how is an ASP-triggered mediator produced in the absence of ASP, and why the generation of an ASP-triggered mediator is not affected by the presence of ASP? Since ASP-acetylated COX-2 generates ATLs from PUFA, we can speculate that minimum amounts of acetylated COX-2 could be naturally present in EVs and the process triggered by an excess of substrate even in absence of ASP. Conversely, if no PUFAs are present, even incorporation of abundant ASP will not trigger the production of ATLs because of the absence of substrate. However, without specific data, this is only guesswork and, clearly, finding answers to these questions require further investigation.

Whereas the incorporation of lipid precursors and pro-resolving mediators by HEI-OC1 EVs was not unexpected given their hydrophobic nature, the amounts were surprisingly high. All these agents were found in concentrations roughly six to seven orders of magnitude higher than the normal values present in human serum [micrograms/ml in HEI-OC1 EVs vs. picograms/ml in human serum (Colas et al., 2014; Dalli et al., 2017)]. Therefore, delivery of very small volumes of EV suspension would be sufficient to reach clinically significant amounts of these pro-resolving mediators in specific organs and tissues. This is particularly important for their intracochlear delivery, given the small size of the auditory organ.

Other very important result is the demonstration that EVs can be loaded simultaneously with different precursors and drugs and that this can be accomplished by simple co-incubation of the EVs with the different agents. As abundantly described in the literature, EVs have been loaded with different molecules either endogenously or exogenously. For instance, mesenchymal and tumor cells incubated with chemotherapeutic drugs, subsequently produced EVs loaded with these drugs (Tang et al., 2012; Pascucci et al., 2014). The main disadvantage of this endogenous approach, however, is that drug incorporation into the cells and into the EVs depends on the particular cells and the mechanisms involved in molecular sorting into EVs, which largely remain to be elucidated. The exogenous approach, which involves loading of EVs after their isolation as described here, is simpler, but not exempt of potential complications. For example, incorporation of hydrophilic agents may be problematic, but even in these situations high loading efficiencies can be obtained with sonication, extrusion or following saponin treatments (Vader et al., 2016).

### S-EVs or L-EVs?

As known from the literature (Mathivanan et al., 2010; Bobrie et al., 2012; Romancino et al., 2013; Zaborowski et al., 2015; Kowal et al., 2016; Xu et al., 2016; Vagner et al., 2019) and confirmed by our results, S-EVs and L-EVs have different composition and, probably, different functions. Since we are interested in using them as nanocarriers of pre-defined molecular cargoes to play specific functions, we wonder whether one of these fractions could be more qualified than the other to be exploited for our therapeutic goal.

One important parameter, the potential amount of cargo able to be incorporated by one fraction or the other, provided some interesting hints. As reported in “Results” section, the total volume and surface (membrane) area of the vesicles present in each fraction are of the same order of magnitude, suggesting that they should be able to incorporate nearly similar amounts of exogenous cargo. However, the already discussed issue of ASP incorporation indicates that this is not necessarily true.

Proteomic results suggest that HEI-OC1 S-EVs, but not L-EVs, contain ANXA1, Gal-1 and Gal-3, known regulators and mediators of inflammation resolution (Kalinec et al., 2017). This result makes S-EVs more attractive as potential nanocarriers in pro-resolving therapies. In addition, they contain many unique proteins that are probably part of the proteome of the parent cells and could perhaps be associated with the function and/or protection of the hearing organ (Kowal et al., 2016; Kalinec et al., 2019). However, dexamethasone can be incorporated as cargo in any EV, and it is already known that this glucocorticoid induces the release of ANXA1 (Kalinec et al., 2009), galectins and other proteins from Hensen cells of the organ of Corti (Kalinec et al., unpublished). Thus, the differences in the proteome of S-EVs and L-EVs could be compensated, at least partially, by the judicious choice of their cargo to include the right molecules and/or pharmacological agents.

Our lipidomic studies showed only small differences between S-Control and L-Control. Most importantly, they showed a huge increase in the variety and amount of lipid metabolites present in EVs of both fractions loaded with AA, EPA, and DHA, erasing any potential difference between S-Control and L-Control (Supplementary Table S3). Although the idea of loading EVs with precursors of pro-resolving mediators, expecting their transformation into lipoxins, resolvins, protectins and/or maresins in the target organ, was very attractive, the observed generation of a number of pro-inflammatory agents inside the EVs makes it inconvenient. In contrast, the fact that L-EVs showed a more efficient incorporation of ASP, LXA4, and RvD1 than S-EVs suggests that larger vesicles could be more effective carriers for anti-inflammatory and pro-resolving agents than smaller ones. Nevertheless, experiments aimed specifically at comparing S-EVs and L-EVs drug delivery to particular organ or cell targets must be performed before arriving at a definitive conclusion. Studies pursuing this goal are currently being performed in our laboratory.

## Conclusion

In the present study, we provide evidence that auditory HEI-OC1 cells generate abundant EVs that can be loaded with combinations of anti-inflammatory drugs and pro-resolving agents in significantly higher concentrations than those normally required for clinical significance. Proteomic and lipidomic studies detected a differential distribution of selected proteins and lipids between small (S-EVs) and large (L-EVS) vesicles. For instance, the S-EVs fraction contains a larger number and diversity of proteins than the L-EVs that could be associated with the involvement of a more efficient cellular mechanism of protein sorting and/or loading. In particular, S-EVs contain the pro-resolving protein mediators ANXA1, Gal-1 and Gal-3 as well as a variety of other molecules that were not found in the larger vesicles. EVs from both fractions can be loaded with anti-inflammatory drugs and pro-resolving mediators, either alone or mixed, making possible the generation of particles with cargoes containing a cocktail of molecules aimed at accelerating inflammation resolution and improving the organ response to inflammation damage. Importantly, since incorporation of PUFAs induces the generation of pro-inflammatory agents in the EVs, which could interfere with the resolution of inflammatory processes, these precursors should not be included as cargo when planning a therapeutic intervention. Therefore, altogether, these results provide support to the idea that EVs from auditory HEI-OC1 cells could be useful nanocarriers for the delivery of anti-inflammatory drugs and pro-resolving mediators, but their cargo should be carefully tailored to ensure that it will indeed promote the successful resolution of inflammatory processes.

## Data Availability Statement

All datasets generated for this study are included in the article/Supplementary Material.

## Author Contributions

GK cultured the cells, isolated EVs, and prepared all the experimental samples. LG, JW, and KF performed proteomic analyses and aspirin incorporation measurements. WC contributed significantly to proteomic analysis and data interpretation. FK designed the study and wrote the manuscript. JW and particularly KF helped edit the manuscript.

## Conflict of Interest

The authors declare that the research was conducted in the absence of any commercial or financial relationships that could be construed as a potential conflict of interest.

## Footnotes

^1^http://www.ucsd-lipidmaps.org
