# Supplementary material for: Extracellular Vesicles From Auditory Cells as Nanocarriers for Anti-inflammatory Drugs and Pro-resolving Mediators
Source: Front Cell Neurosci. 2019 Nov 29;13:530. doi: 10.3389/fncel.2019.00530 (PMC6895008; doi:10.3389/fncel.2019.00530)
Supplement: Supplementary file 4 [file Data_Sheet_1.PDF]

Supplemental Figure #1: Background Curves

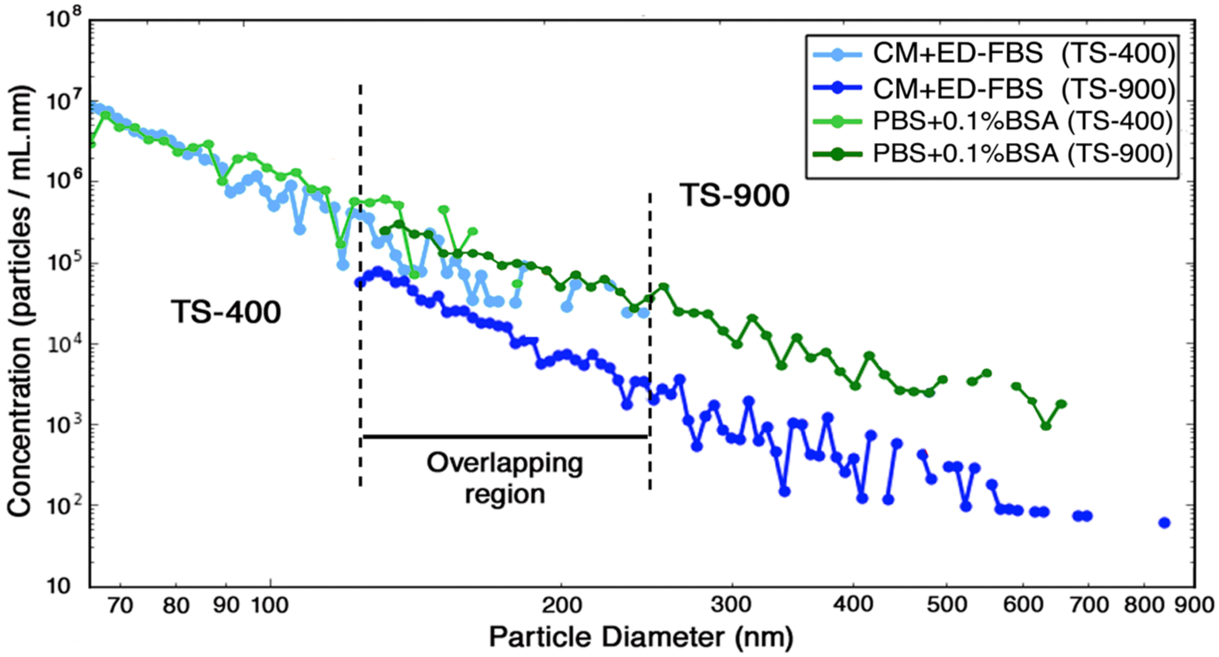

Computer-generated CSD vs. particle size for culture media+exosome-depleted FBS (CM+ED-FBS) and PBS+0.1% BSA in the whole size range. The values for each point of CM+ED-FBS plus those corresponding to PBS+0.1% BSA were considered "Background" and subtracted from the measured values for HEI-OC1 EVs.
